# Supplementary material for: Comprehensive analysis of effect of high-acid-value waste cooking oil on adhesion between aged asphalt and aggregates
Source: PLoS One. 2025 Nov 12;20(11):e0332738. doi: 10.1371/journal.pone.0332738 (PMC12611140; doi:10.1371/journal.pone.0332738)
Supplement: Attachment — Submitted filename: Response to Reviewers.docx [file pone.0332738.s002.docx]

Journal Requirements:

When submitting your revision, we need you to address these additional requirements.

1. Please ensure that your manuscript meets PLOS ONE's style requirements, including those for file naming. The PLOS ONE style templates can be found at

https://journals.plos.org/plosone/s/file?id=wjVg/PLOSOne_formatting_sample_main_body.pdf and

[https://journals.plos.org/plosone/s/file?id=ba62/PLOSOne_formatting_sample_title_authors_affiliations.pdf](https://track.editorialmanager.com/CL0/https:%2F%2Fjournals.plos.org%2Fplosone%2Fs%2Ffile%3Fid=ba62%2FPLOSOne_formatting_sample_title_authors_affiliations.pdf/1/010f019860d2e5dd-3179b67e-8d09-4fa9-8c4c-b99b9668c3b3-000000/SHl0hIQ4uI_2SE8KMaaqInghUDt7tezP_oPCmvcjsls=222)

Thank you for the valuable suggestions provided by the editor. Based on the editor's advice, the article has been revised accordingly.

2. Please note that PLOS One has specific guidelines on code sharing for submissions in which author-generated code underpins the findings in the manuscript. In these cases, we expect all author-generated code to be made available without restrictions upon publication of the work. Please review our guidelines at https://journals.plos.org/plosone/s/materials-and-software-sharing#loc-sharing-code and ensure that your code is shared in a way that follows best practice and facilitates reproducibility and reuse.

Thank you for the valuable suggestions provided by the editor. Based on the editor's advice, the article has been revised accordingly.

3. We note that the grant information you provided in the ‘Funding Information’ and ‘Financial Disclosure’ sections do not match.

When you resubmit, please ensure that you provide the correct grant numbers for the awards you received for your study in the ‘Funding Information’ section.

Thank you for the valuable suggestions provided by the editor. Based on the editor's advice, the article has been revised accordingly.

4. Thank you for stating the following financial disclosure:
“The authors acknowledge support from the Luliang City High-Level Scientific and Technological Talent Introduction Key Research and Development Project (2022RC17), Luliang City high-tech field key research and development projects (2024GX06, 2024GX07), Shanxi Province Science Foundation for Youths Award (202303021222253), and the Innovative Development Plan Project of Shanxi Transportation Research Institute Group Co., Ltd. (22-JKCF-07). “

Please state what role the funders took in the study. If the funders had no role, please state: "The funders had no role in study design, data collection and analysis, decision to publish, or preparation of the manuscript."
If this statement is not correct you must amend it as needed.
Please include this amended Role of Funder statement in your cover letter; we will change the online submission form on your behalf.

Thank you for the valuable suggestions provided by the editor. Based on the editor's advice, the article has been revised accordingly.

5. Thank you for stating the following in the Acknowledgments Section of your manuscript:
“The authors acknowledge support from the Luliang City High-Level Scientific and Technological Talent Introduction Key Research and Development Project (2022RC17), Luliang City high-tech field key research and development projects (2024GX06, 2024GX07), Shanxi Province Science Foundation for Youths Award (202303021222253), and the Innovative Development Plan Project of Shanxi Transportation Research Institute Group Co., Ltd. (22-JKCF-07). “

We note that you have provided funding information that is not currently declared in your Funding Statement. However, funding information should not appear in the Acknowledgments section or other areas of your manuscript. We will only publish funding information present in the Funding Statement section of the online submission form.
Please remove any funding-related text from the manuscript and let us know how you would like to update your Funding Statement. Currently, your Funding Statement reads as follows:
“The author(s) received no specific funding for this work”

Please include your amended statements within your cover letter; we will change the online submission form on your behalf.

Thank you for the valuable suggestions provided by the editor. Based on the editor's advice, the article has been revised accordingly.

6. If the reviewer comments include a recommendation to cite specific previously published works, please review and evaluate these publications to determine whether they are relevant and should be cited. There is no requirement to cite these works unless the editor has indicated otherwise. 

[Note: HTML markup is below. Please do not edit.]

Reviewers' comments:

Reviewer's Responses to Questions

**Comments to the Author**

1. Is the manuscript technically sound, and do the data support the conclusions?

The manuscript must describe a technically sound piece of scientific research with data that supports the conclusions. Experiments must have been conducted rigorously, with appropriate controls, replication, and sample sizes. The conclusions must be drawn appropriately based on the data presented.

Reviewer #1: Yes

Reviewer #2: Yes

2. Has the statistical analysis been performed appropriately and rigorously?

Reviewer #1: No

Reviewer #2: Yes

3. Have the authors made all data underlying the findings in their manuscript fully available?

The [PLOS Data policy](http://track.editorialmanager.com/CL0/http:%2F%2Fwww.plosone.org%2Fstatic%2Fpolicies.action%23sharing/1/010f019860d2e5dd-3179b67e-8d09-4fa9-8c4c-b99b9668c3b3-000000/pvKmdbPuPVX9jlSf7NsktIi4icSARhz5X7x50HLCfEs=222) requires authors to make all data underlying the findings described in their manuscript fully available without restriction, with rare exception (please refer to the Data Availability Statement in the manuscript PDF file). The data should be provided as part of the manuscript or its supporting information, or deposited to a public repository. For example, in addition to summary statistics, the data points behind means, medians and variance measures should be available. If there are restrictions on publicly sharing data—e.g. participant privacy or use of data from a third party—those must be specified.

Reviewer #1: Yes

Reviewer #2: Yes

4. Is the manuscript presented in an intelligible fashion and written in standard English?

PLOS ONE does not copyedit accepted manuscripts, so the language in submitted articles must be clear, correct, and unambiguous. Any typographical or grammatical errors should be corrected at revision, so please note any specific errors here.

Reviewer #1: Yes

Reviewer #2: Yes

5. Review Comments to the Author

Please use the space provided to explain your answers to the questions above. You may also include additional comments for the author, including concerns about dual publication, research ethics, or publication ethics. (Please upload your review as an attachment if it exceeds 20,000 characters)

Reviewer #1: The paper presents valuable findings, but language, figure quality, simulation clarity, and discussion depth must be improved before the manuscript is suitable for publication.

Lines 17–19: "Although the affects of aging on asphalt interface adhesive depend on mineral category..." → Replace “affects” with “effects”.

Thank you to the reviewers for the valuable suggestions. According to the reviewer's opinion, it has been corrected in lines 17 to 19 of the paper.
Lines 94–96: “...which is identical to the lightweight ingredients in virgin asphalt...” change the sentence for better understanding

Thank you to the reviewers for the valuable suggestions. According to the reviewer's opinion, it has been corrected in lines 94 to 96 of the paper.
Lines 156, 165, 428, 579: Multiple instances of awkward phrasing and grammar errors exist throughout the manuscript.

Thank you to the reviewers for the valuable suggestions. According to the reviewer's opinion, it has been corrected in the paper.
Lines 125–136: The literature gap is not very well defined, as the similar work is already done by e.g., Yan et al. 2022; Gao et al. 2019

Thank you to the reviewers for the valuable suggestions. According to the reviewer's opinion, it has been summarize again in the paper.
Figures 3, 5, 7, 8, 10 (Lines ~345–600): The y-axis labels on several graphs (e.g., “Bond strength”, “Work of adhesion”) are too small or unclear. Also, error bars are missing, which are important for interpreting variability across replicates (especially in pull-off strength tests – Fig. 3). Revise figures for better visual clarity and inclusion of standard deviations.

Thank you to the reviewers for the valuable suggestions. According to the reviewer's opinion, figures 3, 5, 7, 8, 10 has been revised in the paper.
Lines 315–320 and 607–609: The units for adhesion work and debonding (mJ/m²) should be consistently formatted. Also, in equations (1)–(6), symbols should be clearly defined immediately after the equation, not included in the paragraphs.
Equations (1)–(6), Lines ~370–590: The formatting of equations in text is inconsistent (e.g., spacing issues, improper symbols).

Thank you to the reviewers for the valuable suggestions. According to the reviewer's opinion, figures 8, 10 and equations (1)–(6) has been revised in the paper.
Lines 525–540 and 566–575: The points about WCO improving asphaltene dispersion and microstructure are repeated.

Thank you very much for the valuable comments provided by the reviewers. Based on their suggestions, we have made corresponding revisions in the paper.d
Lines 660–675: Conclusions are well-structured but could benefit from quantitative mention of key improvements (e.g., % increase in bond strength or adhesion energy after WCO addition).

Thank you to the reviewers for the valuable suggestions. According to the reviewer's opinion, relevant conclusions has been revised in the paper.

Reviewer #2: The article is sound enough for publishing as it expose gap of knowledge and follows good methodology to attain the objectives.
Comments:
1. The introduction must be edited to focus on the problem statement, objectives and research gap. Avoid the general and repeated speech.

Thank you to the reviewers for the valuable suggestions. According to the reviewer's opinion, it has been corrected in the paper.
2. Some figures are pr esented with low quality missing axis. Double check and fix.

Thank you to the reviewers for the valuable suggestions. According to the reviewer's opinion, it has been corrected in the paper.
3. The discussions must be deepened. The results must be justified, verified, and validated.

Thank you to the reviewers for the valuable suggestions. According to the reviewer's opinion, it has been corrected in the paper.
4. The English must be edited.

Thank you to the reviewers for the valuable suggestions. According to the reviewer's opinion, it has been corrected in the paper.
